# Investigation of NS3 Protease Resistance-Associated Variants and Phenotypes for the Prediction of Treatment Response to HCV Triple Therapy

**DOI:** 10.1371/journal.pone.0156731

**Published:** 2016-06-09

**Authors:** Julia Dietz, Daniel Rupp, Simone Susser, Johannes Vermehren, Kai-Henrik Peiffer, Natalie Filmann, Dimitra Bon, Thomas Kuntzen, Stefan Mauss, Georgios Grammatikos, Dany Perner, Caterina Berkowski, Eva Herrmann, Stefan Zeuzem, Ralf Bartenschlager, Christoph Sarrazin

**Affiliations:** 1 Department of Internal Medicine 1, University Hospital Frankfurt, Frankfurt am Main, Germany; 2 Department of Infectious Diseases, Molecular Virology, Heidelberg University, Heidelberg, Germany; 3 Institute of Biostatistics and Mathematical Modeling, Goethe University Frankfurt, Frankfurt am Main, Germany; 4 Klinik für Gastroenterologie und Hepatologie, Universitätsspital Zürich, Zürich, Switzerland; 5 Zentrum für HIV und Hepatogastroenterologie, Düsseldorf, Germany; 6 German Center for Infection Research, Heidelberg University, Heidelberg, Germany; Saint Louis University, UNITED STATES

## Abstract

Triple therapy of chronic hepatitis C virus (HCV) infection with boceprevir (BOC) or telaprevir (TVR) leads to virologic failure in many patients which is often associated with the selection of resistance-associated variants (RAVs). These resistance profiles are of importance for the selection of potential rescue treatment options. In this study, we sequenced baseline NS3 RAVs population-based and investigated the sensitivity of NS3 phenotypes in an HCV replicon assay together with clinical factors for a prediction of treatment response in a cohort of 165 German and Swiss patients treated with a BOC or TVR-based triple therapy. Overall, the prevalence of baseline RAVs was low, although the frequency of RAVs was higher in patients with virologic failure compared to those who achieved a sustained virologic response (SVR) (7% versus 1%, *P* = 0.06). The occurrence of RAVs was associated with a resistant NS3 quasispecies phenotype (*P*<0.001), but the sensitivity of phenotypes was not associated with treatment outcome (*P* = 0.2). The majority of single viral and host predictors of SVR was only weakly associated with treatment response. In multivariate analyses, low AST levels, female sex and an *IFNL4* CC genotype were independently associated with SVR. However, a combined analysis of negative predictors revealed a significantly lower overall number of negative predictors in patients with SVR in comparison to individuals with virologic failure (*P*<0.0001) and the presence of 2 or less negative predictors was indicative for SVR. These results demonstrate that most single baseline viral and host parameters have a weak influence on the response to triple therapy, whereas the overall number of negative predictors has a high predictive value for SVR.

## Introduction

The first DAAs (direct acting antivirals) targeting proteins with critical functions in the HCV replication cycle were the first-generation linear NS3 protease inhibitors (PIs) boceprevir (BOC) and telaprevir (TVR) [1]. Both PIs were approved for treatment of chronic hepatitis C genotype (GT) 1 infection in combination with pegylated (PEG) interferon (IFN)-α and ribavirin (RBV) leading to sustained virologic response (SVR) rates of 67%-75% in treatment-naïve patients [2, 3]. Although the SVR rates were higher in comparison to dual PEG-IFN/RBV treatment, virologic failure was still observed in a substantial number of patients and severe side effects were frequently observed. Furthermore, a selection of resistance-associated variants (RAVs) was detected in many patients at the time of virologic failure [4–6] and these variants were shown to persist for several months [7–9]. Therapies consisting of the administration of BOC or TVR in combination with PEG-IFN and RBV (termed triple therapies) were the standard treatment during 2011 to 2013 in both Europe and the United States and they are currently still used in many countries.

The current new treatment standard uses IFN-free combinations of highly efficient DAAs that lead to very high SVR rates and are associated with few and mostly mild side effects. However, second generation protease inhibitors like simeprevir, paritaprevir or asunaprevir are frequently part of these therapies and overlapping resistance profiles with BOC-/TVR-associated RAVs may significantly impact rescue treatment options after failure of a BOC or TVR-based triple therapy [10]. This study aimed to investigate the impact of baseline NS3 BOC/TVR-associated RAVs together with other clinical factors on virologic treatment outcome. In addition, we evaluated the sensitivity of full patient-derived NS3 quasispecies to BOC/TVR in a phenotypic replicon-based assay for prediction of treatment response.

## Materials and Methods

### Patients

Between 2012 and 2013 a German National non-invasive resistance study investigating baseline resistance (PIRB, PI resistance baseline) was performed at 16 study sites in Germany and one site in Switzerland. Inclusion criteria for the study were a chronic hepatitis C GT1 infection, a treatment-naïve status or a pretreatment with (PEG)-IFN +/- RBV and a planned triple therapy with BOC or TVR. The exclusion criterion was an infection with GT2, 3, 4, 5 or 6. Caucasian patients were treated in a real-world setting with BOC or TVR in combination with PEG-IFN-α/RBV according to the label and serum and EDTA blood were collected from 196 patients before initiation of triple therapy. Epidemiological, biochemical, virological and histological parameters were documented. The outcome of triple therapy was classified as follows 1) SVR (negative HCV RNA 12 weeks after the end of treatment), 2) non-response (TVR: HCV RNA >1000 IU/mL at week 4 or 12, positive HCV RNA week 24. BOC: HCV RNA >100 IU/mL week 12, positive HCV RNA week 24. BOC and TVR: no negative HCV RNA measurement throughout the entire therapy), 3) non-response including breakthrough (achievement of negative HCV RNA followed by an HCV RNA increase of >1000 IU/mL or >1log10 during therapy), 4) relapse (achievement of negative HCV RNA at the end of treatment followed by positive HCV RNA post-treatment) [11]. Patients who discontinued treatment due to non-virologic reasons and patients who were lost to follow up were excluded (n = 31). Thus, 165 patients were available for the present analysis. The study was performed according to the Declaration of Helsinki and approval for the enrollment and the utilization of serum and EDTA blood samples for research purposes was obtained from the local ethics committee (Ethik-Komission der J. W. Goethe-Universität Frankfurt) and written informed consent including genetic testing was received from all patients.

### NS3 protease amplification, sequencing and analysis of RAVs

HCV RNA extraction and amplification of the NS3 protease encoding region were done as previously described [12]. Briefly, 140 μl serum was used for RNA extraction and cDNA was produced with 8 μl RNA and SuperScript III Reverse Transcriptase (Invitrogen). Nested NS3 PCR sequence-specific amplifications were performed with 1/10 of cDNA or outer PCR product, respectively by using the Fast Cycling PCR Kit (Qiagen, Hilden, Germany) and primers and cycling conditions as published [13, 14]. Resulting PCR products were gel-purified using the QIAquick Gel Extraction Kit (Qiagen, Hilden, Germany). Population-based sequencing was conducted with the inner forward PCR primer using the Big Dye Terminator v3.1 Cycle Sequencing Kit according to the manufacturer´s protocol on an ABI Prism 3130*xl* Genetic Analyzer (Applied Biosystems, Foster City, CA, USA). Obtained sequences encompassing the 543 bp NS3 protease region (NS3 amino acids 1–181) were proofread and aligned using BioEdit version 7.2.3 [15]. RAVs were considered as relevant if they were described to be associated with treatment failure *in vivo* and/or if they had been demonstrated to confer a greater than 2-fold change of susceptibility to BOC/TVR in comparison to a wildtype reference strain *in vitro* assays [10]. RAVs were analyzed at the following positions each in comparison to the respective HCV reference strain: (GT1a: H77; GT1b: Con1): V36A/M, T54A/S, R155G/I/K/T/Q, A156S/T/V (positions relevant for BOC and TVR). Additional RAVs with relevance only for BOC were investigated at positions V55A, V158I, V170T and M175L (GT 1b). The sensitivity of population-based sequencing is approximately 20% for minority variants. Based on previous reports [14, 16], all RAVs at the respective positions in the electropherogram were considered which also included minority variants which were detected as mixed peaks in the sequence. HCV subtypes were determined based on HCV nucleotide sequences and NS3 protease sequences were denoted as wildtype when no RAVs according to the definition outlined above were detected.

### Patient selection and construction of patient-derived NS3 replicon libraries for phenotyping

For the phenotyping of NS3 quasispecies variants, we used 33 patients with virologic failure available in July 2014 and randomly selected 51 patients with SVR out of a study population of 81 patients with SVR available at that time. Random selection used a randomization list calculated with the Matlab software package (MathWorks, Natick, MA, USA) and no significant differences were detected between the patients selected for phenotyping and the remaining individuals of the cohort. Including a patient number of 33 versus 51 individuals allowed a power above 85% to detect differences between the groups using a Wilcoxon-Mann-Whitney-U-test with a significance level of 5% if the Mann-Whitney estimator was at least 0.7.

For the construction of replicon libraries, the HCV GT 1b subgenomic replicon pFKi341-PiLucNS3-3'_ET [17] was modified as reported previously and ClaI and AscI sites were inserted into the linker region of the NS3- protease and helicase-coding region allowing the shuttling of NS3-protease specific amplicons obtained with patient sera [18]. Patient NS3 inserts were generated by performing PCR reactions with primers published elsewhere using the NS3 PCR product that was used for sequence analysis [18]. PCR mixtures were incubated at 95°C for 10 minutes, followed by 30 cycles as follows: 95°C for 30 seconds, 60°C for 30 seconds and 72°C for 1 minute. A final elongations step was performed at 72°C for 7 minutes. Purified PCR products were digested using ClaI and AscI, inserted into pFKi341-PiLucNS3-3'_ET and amplified in *E*. *coli* cells. Ten percent of the transformation mixture was plated onto LB-Agar with 100 μg/mL ampicillin to determine the ligation efficiency by comparison with a ligation reaction without insert. The remaining aliquot of cells was grown in LB medium containing ampicillin, followed by the plasmid isolation using the NucleoSpin Plasmid kit according to the manufacturer's protocol (Macherey-Nagel, Düren, Germany). Additionally, ligation was quality controlled by restriction digestion of the obtained library and by bulk sequencing to ensure the insertion of patient derived NS3 protease sequences. The parental pFKi341-PiLucNS3-3'_ET construct served as reference for GT1b patient samples. To generate a reference for GT1a patients, the NS3 protease from the H77 reference strain was amplified from pFKi341-PiLucH77S [19] and inserted into pFKi341-PiLucNS3-3'_ET as described above.

### *In vitro* transcription and electroporation of subgenomic HCV RNAs

Ten microgram plasmid DNA was linearized with PvuI, purified and used for *in vitro* transcription as specified in S1 File. Huh7-Lunet cells [20] were trypsinized, washed with PBS and resuspended at a concentration of 1x10^7^ cells/mL in Cytomix [21] supplemented with 2 mM ATP and 5 mM glutathione. RNA (2.5 μg) was mixed with 100 μl cell suspension and electroporated into Huh/-Lunet cells using a GenePulser system (Bio-Rad, Hercules, CA, USA) at 500 μF and 166 V. Cells were immediately diluted in complete DMEM containing 10% FCS, 1% penicillin, 1% streptomycin as well as 1% non-essential amino acids and seeded at a density of 1.5 x 10^4^ cells/well.

### Titration of boceprevir and telaprevir; luciferase assay and data analysis

BOC and TVR were dissolved in DMSO and serially diluted to final concentrations of 0–3.3 μM; DMSO concentration was adjusted to 0.05% (v/v) in all concentrations. Four hours after electroporation, for each patient library an aliquot of the cells was harvested to determine the transfection efficiency by measuring the luciferase activity (see S1 File) and BOC or TVR were added to the remaining cells that were incubated for further 68h. Based on relative light units (RLU) which were calculated as the mean of at least three independent experiments (each experiment was performed in triplicates), IC50 values for BOC and TVR were calculated by non-linear regression curve-fit using the GraphPad Prism software package (version 5.00; GraphPad Software, La Jolla, CA, USA). The mean IC50 value was then normalized to the IC50 values of the corresponding reference construct for each genotype and expressed as mean fold-change IC50 value. The replication fitness of each patient library was expressed as the ratio of the 72h/4h RLU and describes the increment of firefly luciferase signal over the value measured 4h after electroporation.

### *IFN-Lambda4 (IFNL4)* single nucleotide polymorphisms genotyping

Genomic DNA of patient samples was extracted from EDTA blood using the QIAamp DNA Blood Kit (Qiagen, Hilden, Germany). A real-time polymerase chain reaction was conducted for SNP genotyping rs12979860 (formerly known as *IL28B*) using a StepOnePlus instrument (Applied Biosystems Foster City, CA, USA) as described previously [22, 23].

### Quantification of IP-10 (interferon-γ-inducible-protein-10) levels

IP10 levels were measured in all baseline samples using the commercially available CXCL10/IP-10 Quantikine ELISA Kit according to the manufacturer´s protocol (R&D Systems, Minneapolis, MN, USA) and 75μl of 1:4 diluted serum samples.

### Analysis of negative predictors

Adapted on previous studies [10, 11, 22, 24–28], we considered the following factors as negative predictors for treatment response: *IFNL4* non-CC genotype, HCV subtype 1a, a high HCV viral load, high IP10 levels, the presence of cirrhosis, the presence of BOC/TVR-associated RAVs, resistant phenotypes (>2-fold change). Based on results obtained in our cohort, cut-offs for the HCV viral load and IP10 levels were calculated. Therefore the median HCV viral load and IP10 level were calculated for the groups of patients with virologic failure versus SVR. A further calculation of the mean value using both medians resulted in a cut-off >1,971,750 IU/mL (viral load) and >428 pg/mL (IP10) which were used as negative predictors. Finally, for each patient the number of negative predictors present was analyzed.

### Statistics

Statistical analyses were conducted using BIAS (BIAS for Windows, Version 10, Epsilon Verlag, Darmstadt, Germany). For the investigation of continuous variables (e.g. HCV viral load, IP10 levels, phenotype sensitivity, number of negative predictors), the Wilcoxon-Mann-Whitney-U-test (comparison of 2 groups) or the Kruskall-Wallis test (3 or more groups) were conducted. For an investigation of dichotomic variables (e.g. *IFNL4* genotype, HCV GT, cirrhosis, prior null response, NS3 RAVs) we applied contingency tables (Chi-square test, Fisher´s exact test if there were expected frequencies less than 5). A correlation of two parameters (fitness and IC50 fold change) was calculated using Spearman´s rank correlation. To analyze the influence of two factors on one parameter (e.g. cirrhosis and treatment outcome on phenotype sensitivity), a bifactorial rank analysis was conducted. To identify independent predictors of SVR, a multivariate logistic regression analysis was performed including all significant parameters from the univariate analysis and quantitative variables were transformed logarithmically. *P*-values of less than 0.05 were considered significant. For the investigation of different cut-off levels for the prediction of SVR, we performed a ROC analysis and used the Youden criterion for cut-off selection.

## Results

### Baseline patient characteristics

The baseline parameters of all 165 patients are displayed in Table 1. Slightly more than half of the patients (n = 91, 55%) was infected with subtype 1b and the median HCV viral load was 1.6x10^6^ IU/mL. The favorable *IFNL4* CC genotype had 29% (n = 46) of patients. A liver cirrhosis was detected in 29% (n = 47) of individuals and 63% (n = 103) were treatment-experienced (PEG-IFN/RBV, IFN/RBV or IFN-monotherapy). The majority (n = 136, 82%) was treated with a TVR-based triple therapy. The overall SVR rate of patients receiving a BOC or TVR-based triple therapy was 73% (n = 121). In separate analyses, the SVR rate for TVR-treated patients was 78% (n = 106) and for BOC-treated individuals 52% (n = 15).

**Table 1:** Baseline patient characteristics.

Parameter			Patients (n = 165)
Age (years) [median (range)]			52 (29–74)
Sex			
	Male [n (%)]		105 (63.6%)
	Female [n (%)]		60 (36.4%)
Body mass index (kg/m^2^) [median (range)]			24.6 (18.4–48.4)
*IFNL4* genotype			
	CC [n (%)]		46 (29.1%)
	non-CC [n (%)]		112 (70.9%)
	n.d. [n]		7
Liver enyzmes			
	ALT (U/l) [median (range)]		86 (19–551)
	AST (U/l) [median (range)]		65 (5–301)
	GGT [median (range)]		82 (12–508)
Cirrhosis			
	Patients with cirrhosis [n (%)]		47 (28.5%)
	Patients without cirrhosis [n (%)]		118 (71.5%)
Fibrosis			
	F0-F2 [n (%)]		40 (42.1%)
	F3-F4 [n (%)]		55 (57.9%)
	n.d. [n]		70
HCV infection			
	HCV viral load (IU/mL) [median (range)]		1.6x10^6^ (3.6x10^3^–3.6x10^7^)
	HCV GT 1a [n (%)]		74 (44.8%)
	HCV GT 1b [n (%)]		91 (55.2%)
Current Treatment			
	BOC-based [n (%)]		29 (17.6%)
	TVR-based [n (%)]		136 (82.4%)
Outcome			
	SVR [n (%)]		121 (73.3%)
	Virologic Failure [n (%)]		44 (26.7)
		Non-Response [n (% virologic failure)]	14 (31.8%)
		Breakthrough [n (% virologic failure)]	12 (27.3%)
		Relapse [n (% virologic failure)]	18 (40.9%)
Treatment History			
	Treatment-naïve [n (%)]		60 (36.8%)
	Treatment-experienced ((PEG)-IFN +/- RBV) [n (%)]		103 (63.2%)
	n.d. [n]		2
HIV Coinfection			
	Patients with HIV infection [n (%)]		11 (7.9%)
	Patients with HIV <20 copies/mL [n% HIV infected)]		10 (90.9%)
	HIV status n.d.		26
HBV Coinfection			
	Patients with HBV infection [n (%)]		2 (1.4%)
	Patients with HBV <20 copies/mL [n% HBV infected)]		2 (100%)
	HBV status n.d.		26

### Baseline NS3 protease RAVs

**Fig 1:**
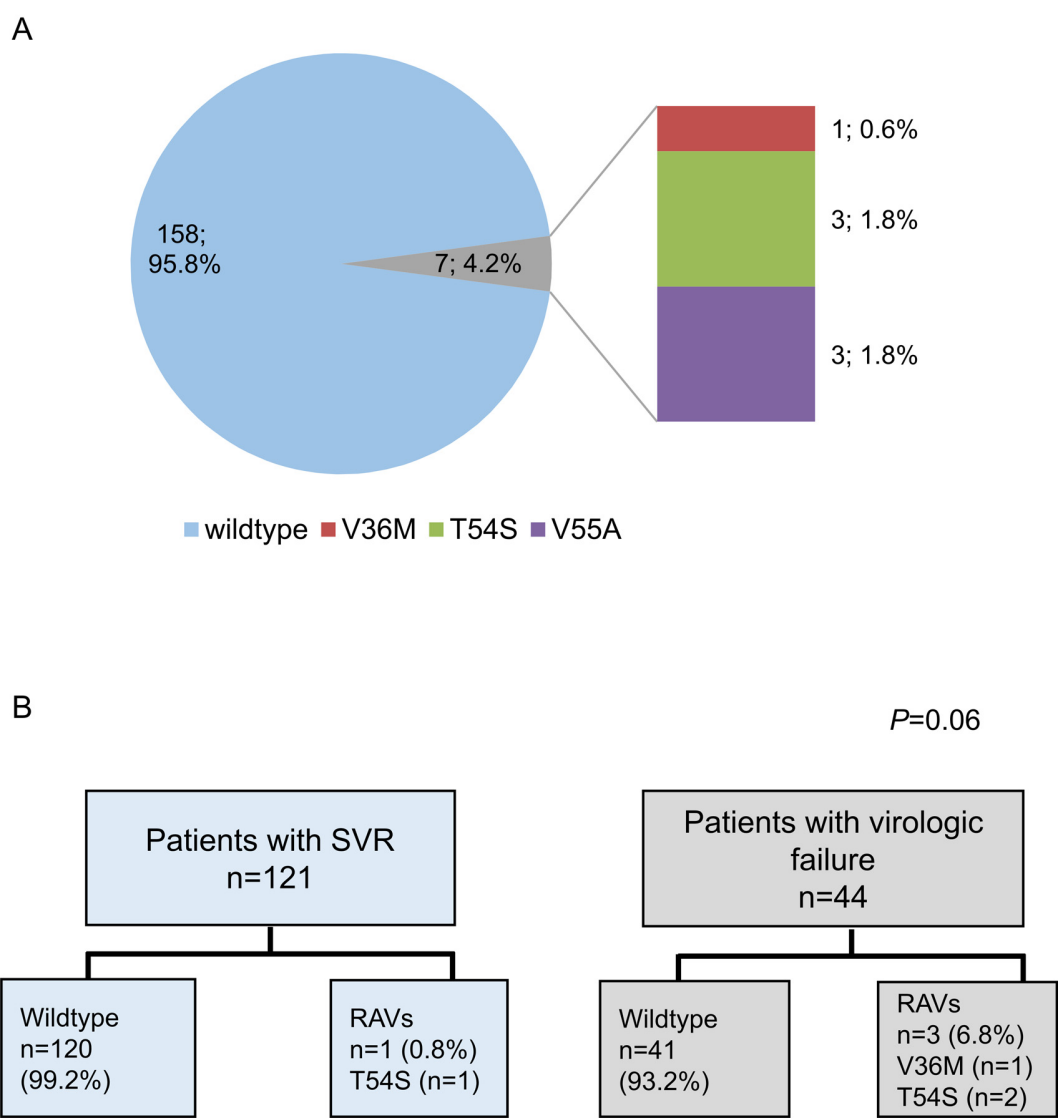
A) Prevalence BOC/TVR-relevant NS3 RAVs at baseline. B) Prevalence of RAVs in patients with SVR versus virologic failure (without V55A variant, see text).

We investigated NS3 protease sequences at several positions which had been shown to be relevant for the emergence of RAVs. At baseline, RAVs were rarely detected. The most prevalent RAVs were T54S and V55A (n = 3 each, 1.8%), followed by V36M (n = 1, 0.6%) (Fig 1A). T54S was found only in subtype 1b and V36M in subtype 1a, whereas V55A was detected in two patients with subtype 1a and one patient with subtype 1b. As the V55A variant causes only resistance to BOC but was detected at baseline exclusively in patients who were later treated with TVR, this variant was excluded from further analyses (Fig 1B). All detected RAVs were low-level resistant to BOC and TVR. While the frequency of RAVs was higher in patients with virologic failure compared to SVR (n = 3, 7% versus n = 1, 1%), this difference was not significant (*P* = 0.06).

### NS3 phenotypes in correlation with viral and host factors as well as with the outcome of therapy

**Fig 2:**
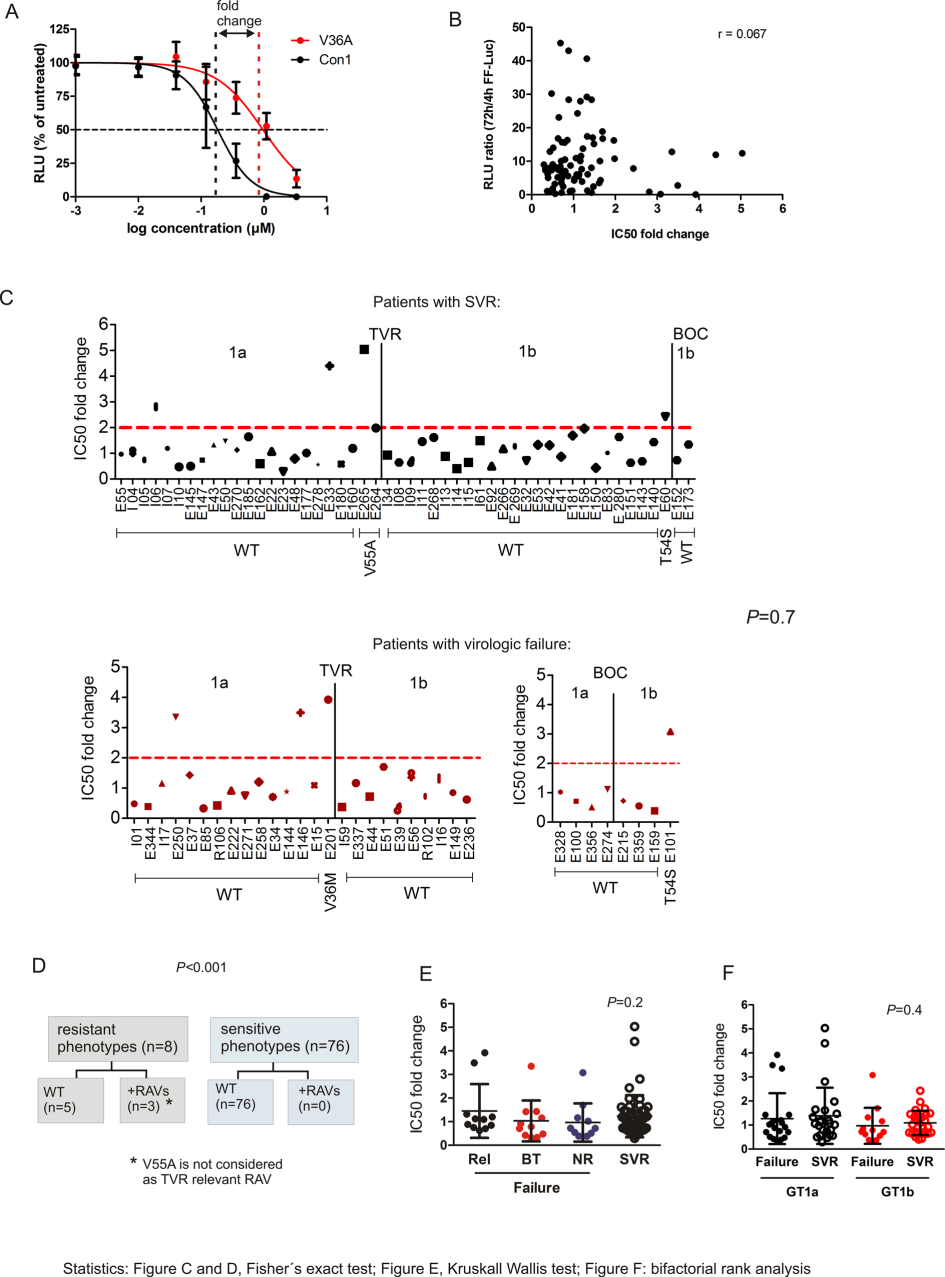
BOC/TVR sensitivity of baseline NS3 protease subgenomic HCV replicon libraries. A) Exemplary representation of IC50 value determination and fold change calculation using titration curves of the wildtype GT1b reference strain (Con1) and the corresponding strain with the low level resistance-conferring RAV V36A. B) Representation of the replication fitness of NS3 subgenomic HCV replicons which is defined as the 72h/4h luciferase ratio relative to the apparent drug sensitivity expressed as fold change of the IC50 value. (c) Sensitivity of single phenotypes in correlation with RAVs and outcome of infection. (d) Association of phenotype sensitivity with RAVs. (e) Phenotype sensitivity after subgrouping of virologic failure samples by relapse (Rel.), breakthrough (BT) and non-response (NR) in comparison to SVR. (f) Subgrouping of NS3 phenotypes according to the viral subtype.

The sensitivity of the patient NS3 quasispecies towards BOC/TVR was investigated using a replicon based assay and IC50 values were calculated out of BOC/TVR titration curves as a measure for drug sensitivity (Fig 2A). We considered phenotypes as resistant, if the IC50 value was at least 2-times increased in comparison to the wildtype genotype control. For all further analyses those IC50 values were taken into account that were generated *in vitro* in presence of the same PI also used *in vivo*. We found no correlation of the IC50 fold change value with the replication fitness of NS3 libraries (72h/4h ratio) (r = 0.067, Fig 2B). Resistant as well as sensitive phenotypes were detected in patients with virologic failure and in individuals with SVR (Fig 2C, *P* = 0.7). Similar to the prevalence of RAVs, the frequency of resistant phenotypes was low (n = 8/84, 9.5%) and the occurrence of RAVs was associated with a resistant phenotype (Fig 2D, *P*<0.001). Subgrouping of samples with virologic failure by relapse (Rel), breakthrough (BT) and non-response (NR) showed no differences compared to patients with SVR (Fig 2E, *P* = 0.2). A slightly increased occurrence of resistant phenotypes was found in patients with subtype 1a, but no difference between samples from patients who failed antiviral therapy and patients with SVR was detected (Fig 2F, *P* = 0.4).

**Fig 3:**
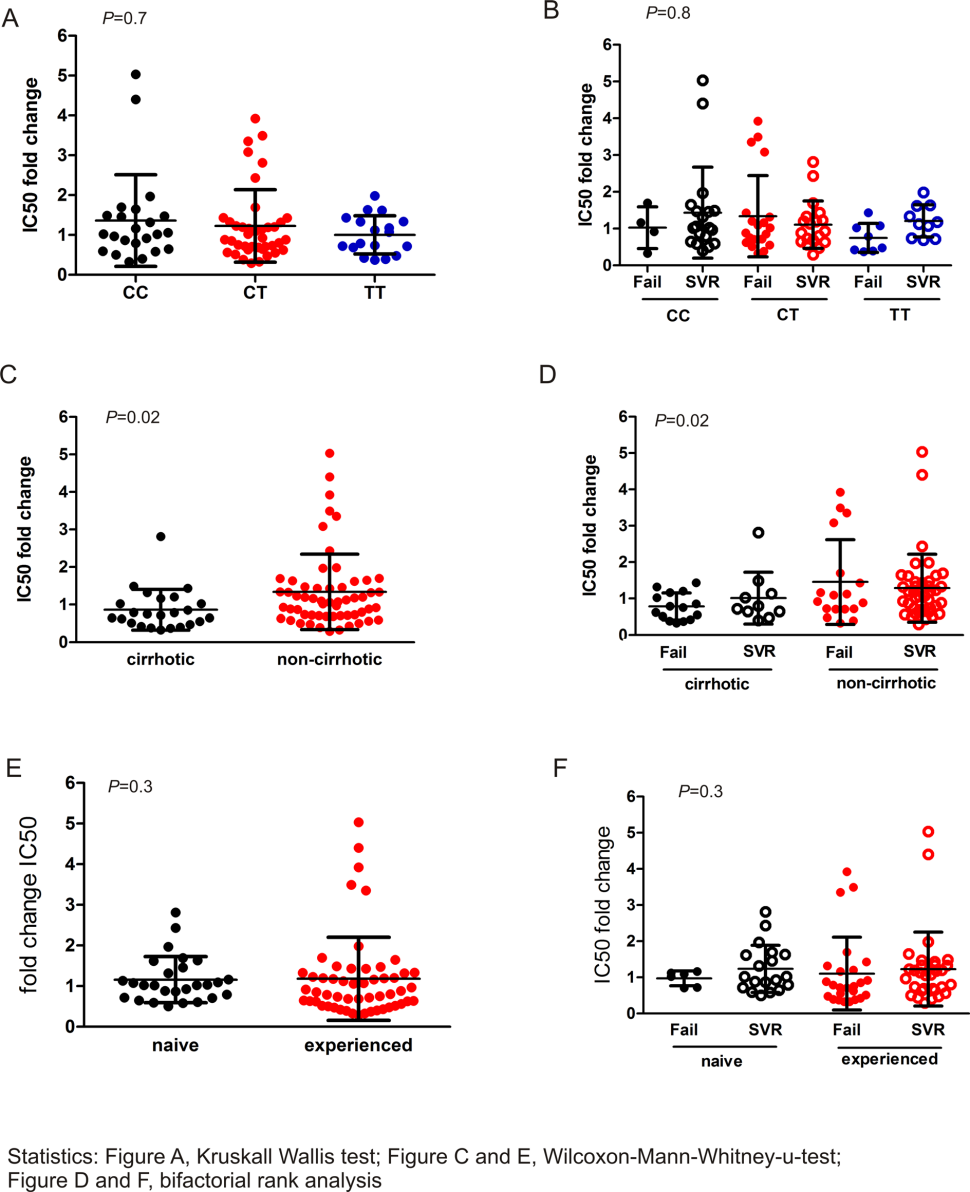
NS3 phenotype sensitivity in correlation with host factors. A, B) NS3 phenotypes and patient *IFNL4* genotype. C, D) NS3 phenotypes and the presence of liver cirrhosis. E, F) Treatment history and NS3 phenotype sensitivity.

Next, a possible influence of host factors on the sensitivity of viral phenotypes was investigated. We observed more resistant phenotypes in patients with *IFNL4* CC and CT genotypes compared to TT. However, phenotype sensitivity was not significantly associated with *IFNL4* after subgrouping for the outcome of therapy (Fig 3A, *P* = 0.7 and 3B, *P* = 0.8). Interestingly, NS3 replicon libraries from patients with cirrhosis showed significantly lower IC50 fold change values in comparison to non-cirrhotic individuals (Fig 3C and 3D, *P* = 0.02 each). The treatment history had no influence on the sensitivity of NS3 protease libraries (Fig 3E and 3F, *P* = 0.3 each).

### Predictors of treatment outcome

In univariate analyses several baseline parameters were significantly associated with SVR (Table 2). In a multivariate logistic regression model, we identified low AST levels, female sex and *IFNL4* CC genotype as remaining independent predictors of SVR (*P*<0.05). Among these, a low AST level was the factor with a high predictive value for SVR (P<0.0001).

**Table 2:** Uni- and multivariate analysis of baseline predictors of SVR to HCV triple therapy.

Patient characteristics	Univariate analysis			Multivariate analysis	
	continuous variables in [median (range)]				
	Virologic Failure	SVR	*P*-value	OR (95% CI)	*P*-value
	n = 44	n = 121			
Age (years)	50 (33–66)	50 (29–74)	0.8*		
BMI (kg/m^2^)	26.6 (21.1–28.4)	25.1 (18.4–36.3)	0.6*		
Female sex [n (%)]	9 (20.5%)	51 (42.2%)	0.02***	3.7994 (1.3474–10.7181)	0.01
ALT [times upper limit of normal]	2.9 (0.7–11.0)	1.7 (0–12.8)	0.0007*		
AST [times upper limit of normal]	2.7 (0.6–7.5)	1.4 (0.1–6.7)	<0.0001*	53.4759 (7.8616–357.1429-)	<0.0001
GGT [times upper limit of normal]	2.8 (0.3–10.3)	1.2 (0.2–8.8)	<0.0001*		
Bilirubin [mg dL-1]	0.6 (0.1–3.5)	0.5 (0.2–2.1)	0.09*		
HCV viral load [IU/mL]	2.6x10^6^	1.4x10^6^	0.1*		
IP10 [pg/mL]	457 (122–2941)	398 (94–2001)	0.02*		
*IFNL4* rs12979860 CC/non-CC [n (%)]	6/44 (13.6%)	40/114 (35.1%)	0.01***	4.7483 (1.4824–15.1976-)	0.009
HCV subtype 1b [n (%)]	20 (45.5%)	71 (58.7%)	0.2***		
Prior Null Response [n (% pretreated patients)]	9/28 (32.1%)	11/67 (16.4%)	0.2***		
Liver cirrhosis [n (%)]	50%	20.7%	0.0005***		
NS3 RAVs [n (%)]	3 (6.8%)	1 (0.8%)	0.06**		
NS3 phenotypes [IC_50_ fold change]	0.8501 (0.3279–3.9233)#	1.0200 (0.2927–5.0340)#	0.2*		
NS3 phenotypes >2-fold change	4/33 (12.2%)	4/51 (7.8%)	0.7**		

*P* values of univariate analyses are uncorrected for multiple testing

*Wilcoxon Mann Whitney U Test

**Fisher exact test for n<20

***Chi-square test

# NS3 phenotyping was performed in a subgroup of patients (n = 33, virologic failure; n = 55, SVR)

OR: Odds ratio, 95% CI, 95% confidence interval

### Combined analysis of predictors of treatment outcome

**Fig 4:**
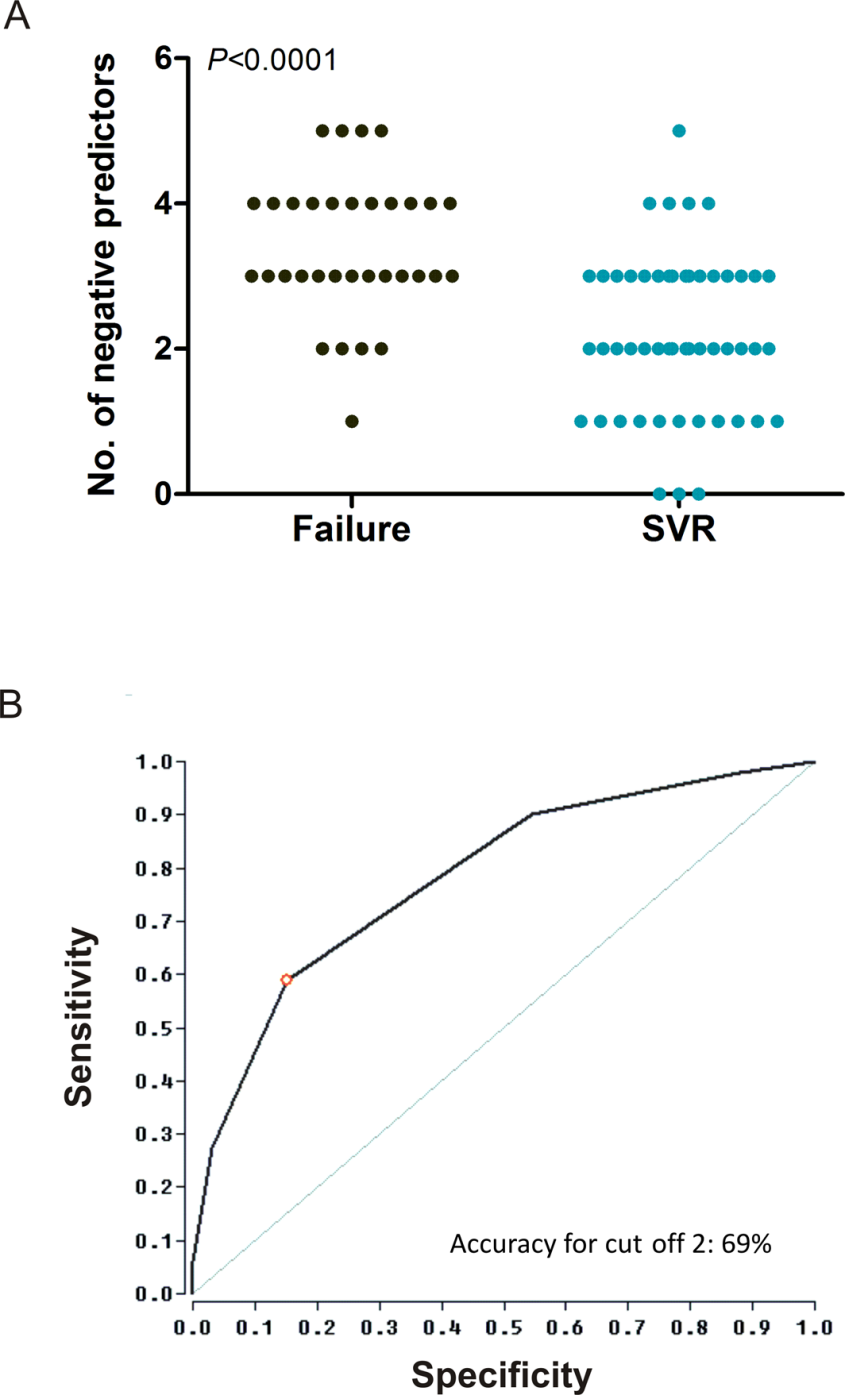
Analysis of negative predictors of treatment response. A) Scatter plot of negative predictors stratified by treatment outcome. Several negative predictors known from the literature (*IFNL4* non-CC genotype, HCV subtype 1a, high HCV viral load and IP10 levels, the presence of cirrhosis, BOC/TVR-resistant RAVs and phenotypes) were investigated in a subgroup of patients with available NS3 phenotypes. B) ROC analysis for defining a cut-off for the number of negative predictors. Thereby, 2 or less negative predictors correspond to a specificity of 85% and a sensitivity of 59% for predicting SVR.

We analyzed the number of negative predictors known from the literature (see material and methods) in the study cohort and compared the number of negative predictors present in patients with virologic failure versus SVR. For this subanalysis, we only included patients for which NS3 phenotypes were determined. Interestingly, the overall number of negative predictors was lower (median 2.0, range 0–5) in individuals with SVR versus patients with virologic failure (median 3.0, range 1–5) and this was highly significant (Fig 4A, *P*<0.0001).

A ROC analysis identified the presence of 2 or less negative predictors as indicative for an SVR and 3 or more negative predictors were indicating a virologic failure (Fig 4B). The PPV (positive predictive value), NPV (negative predictive value), sensitivity and specificity as well as accuracy for the prediction of SVR using a cut-off level of 2 or less are listed in Table 3.

**Table 3:** Predictability of SVR using a cut-off level of 2 or less negative predictors

PPV [%]	30/35 (86%)
NPV [%]	28/49 (57%)
Sensitivity [%]	30/51 (59%)
Specificity [%]	28/33 (85%)
Accuracy [%]	58/84 (69%)

## Discussion

For the success of a triple therapy with BOC/TVR, at least a partial response towards PEG-IFN/RBV is of central importance, as otherwise there is a risk of a functional PI monotherapy. Therefore, baseline factors that influence PEG-IFN/RBV response may also have an effect triple therapies with BOC or TVR [11]. In the past, multiple baseline predictors of response to IFN-based therapies were identified. These include host factors such as a younger age (<45 years), a lower BMI, the absence of advanced fibrosis/cirrhosis, a naïve treatment status and an *IFNL4* CC genotype. Viral parameters favoring SVR are a low baseline viral load and HCV genotypes 2 and 3 [11, 22, 24–26].

The impact of baseline RAVs on the outcome of DAA-based therapies is discussed controversially. Before treatment initiation, NS3 RAVs are detectable at different frequencies; however RAVs are selected rapidly during PI treatment which may have the consequence of a viral breakthrough and treatment failure [16, 29, 30]. To investigate the influence of NS3 RAVs and phenotypes as well as other clinical factors on the outcome of triple therapy, we performed this study in a cohort of 165 patients treated with a BOC or TVR-based triple therapy. In accordance with other studies [4, 16, 28, 31], the overall prevalence of BOC/TVR-relevant RAVs was low at baseline (4.2%) and all observed RAVs were low-level resistant to both substances. Most frequent were T54S and V55A (each 1.8%), followed by V36M (0.6%) (Fig 1A). The prevalence of V36M in subtype 1a is in good accordance with described frequencies of 0.2%-0.6% in the literature [16, 28, 31]. T54S in subtype 1b was detected at frequencies of 1.2%-2% [16, 28, 31] which is also in line with our own observations. There was a trend to higher frequencies of baseline RAVs in patients with virologic failure versus SVR (Fig 1B), but as the overall number of patients was rather low and baseline RAVs were uncommon, this analysis was not statistically powered to detect an impact of baseline RAVs which could limit this study. That pre-existing RAVs are not solely determining the outcome of triple therapy was expectable and was also shown in previous reports [4, 28, 32, 33]. Another limitation of the study could be that deep sequencing for the detection of minor NS3 RAVs was not performed. However, it seems that a deep sequencing analysis of RAVs is not superior to population-based sequencing with respect to the prediction of treatment response [33, 34].

Next, NS3 phenotypes were investigated in correlation with the outcome of triple therapy and other viral and host factors. For the generation of patient-derived NS3 libraries; LB medium was directly inoculated with the transformation mixture without prior selection of one clone which would represent only one NS3 sequence. Therefore, this approach enables to study the sensitivity of the NS3 quasispecies. As the fitness of NS3 replicon libraries correlated not with the BOC/TVR sensitivity, sensitive phenotypes are presumably not caused by a reduced replication fitness (Fig 2B). The occurrence of resistant phenotypes was associated with the detection of RAVs and all patients with RAVs displayed a resistant phenotype. Interestingly, in some patients with resistant phenotypes no RAVs were detected by population-based sequencing (Fig 2C and 2D). We cannot rule out that minor NS3 variants contributed to phenotype sensitivity, but as the clinical relevance of minor RAVs is not verified so far (see above), we assume that this is a rather weak effect. Furthermore, the clinical parameters as well as additional NS3 protease variations (besides the positions analyzed for RAVs) were not markedly different in these individuals compared to the remaining patients of the cohort. However, in a recent publication several mutations within the NS3 helicase were identified which enhanced or also impaired HCV replication [35]. We only incorporated NS3 protease sequences in the HCV replicon for phenotyping. One speculation could be that the unusual phenotypes are less replication competent in their natural HCV backbone, possibly due to mutations in the NS3 helicase which impair HCV replication. To investigate this, the NS3 helicase region of all patients would have to be sequenced.

Regarding the outcome of triple therapy, no influence of NS3 phenotypes on treatment response was detected (Fig 2E). Concerning the correlation of phenotypes with host factors, no association of the NS3 phenotype with the *IFNL4* genotype or the pretreatment status was found (Fig 3A, 3B, 3E and 3F). Interestingly, resistant phenotypes were significantly more prevalent in patients with a non-cirrhotic liver (Fig 3C and 3D). Several studies showed that cirrhosis is associated with a dysfunction of the immune system [36]. Furthermore, at baseline the occurrence of RAVs was higher in patients with cirrhosis compared to individuals without cirrhosis [10, 37], which seems to contradict our findings. Cirrhosis is accompanied by a systemic inflammation leading to an immune cell stimulation [36] and it may be conceivable that these activated cells lead to a survival of sensitive phenotypes which could probably adapt more quickly to the changing host environment.

Besides NS3 RAVs and phenotypes, several additional viral and host factors were investigated with respect to their influence on the response to triple therapy. In univariate analyses lower liver enzymes and IP10 levels, female sex, *IFNL4* CC genotype and the absence of cirrhosis were all associated with SVR. In multivariate analyses low AST levels, female sex and an *IFNL4* CC genotype were confirmed as independent predictors for SVR (Table 2). This is in line with large clinical studies which also identified *IFNL4* CC genotype and the stage of fibrosis as predictors for SVR [2, 3]. Furthermore, females have long been regarded as "easy-to-treat" and are more likely to clear an acute hepatitis C infection [10, 38]. In another study, low AST levels were also identified as predictor for SVR in patients infected with HCV genotype 4 [39]. However, the stage of liver fibrosis is a more feasible measure for the functionality of the liver for the prediction of treatment response in comparison to liver enzyme levels. Unfortunately, in this real world study the precise stage of fibrosis was not determined in most of study centers which precludes this analysis.

As the majority of baseline predictors correlated only weakly with treatment outcome, the response to triple therapy may be influenced by a combination of several negative predictors. We investigated the prevalence of negative predictors described in the literature in the present cohort. Interestingly, the overall number of negative predictors was significantly lower in patients with SVR versus virologic failure and a cut-off of 2 or less negative predictors was indicative for an SVR (Fig 4A and 4B). Also for IFN-free DAA combination regimens (for example SMV plus SOF or LDV plus SOF) single negative predictors, like baseline RAVs, seem to be only of importance if other stress factors are existing at the same time like the presence of liver cirrhosis or a shortened treatment duration [10]. However, in the present study, some patients with unusual characteristics were identified and the range of negative predictors was relatively high in both groups. Patients who achieved an SVR despite the presence of 4 or more negative predictors (n = 5) had a low BMI and low liver enzyme levels, were treatment-naïve or had an eRVR (extended rapid virological response) indicating inherent sensitivity to IFN-α [2, 3]. Only one individual experienced a virologic treatment failure despite having only one negative predictor (*IFNL4* non-CC genotype). This is probably due to an extremely high BMI of >45 which is associated with a potential underdosing of medication [40].

Nevertheless, another limitation of this study could be that PI-based triple therapies are not of major importance for the treatment of chronic hepatitis C anymore. However, some countries follow the policy that only patients who are difficult-to-treat are able to receive an IFN-free treatment. Individuals in an early stage of disease without any comorbidities should first wait or a PI-based triple therapy is offered to these patients. Moreover, the conduction of phenotypic analyses is also of importance for the investigation of resistance to all-oral HCV therapies. While pre-existing RAVs within NS3 are rare and especially the replication of high-level resistant variants is reduced, this is not the case for NS5A and non-nucleoside NS5B RAVs. Here, RAVs at baseline are frequently found without an impairment of viral replication. It is therefore reasonable that a phenotyping of the HCV quasispecies will be of more importance for patients treated with NS5A and non-nucleoside NS5B inhibitors.

In conclusion our findings show a slightly higher prevalence of baseline RAVs in patients with virologic failure versus individuals with SVR, although this was not significant. Interestingly, the occurrence of RAVs correlated with a resistant NS3 phenotype in a replicon-based assay. Single baseline viral and host parameters were only weakly associated with the treatment response to triple therapy. In multivariate analyses *IFNL4* genotype, sex and AST levels turned out as independent predictors of SVR. Finally, the overall number of negative predictors was significantly lower in patients with SVR and the presence of 2 or less negative predictors was highly predictive for SVR.

## Supporting Information

S1 File NS3 phenotyping (DOCX)
