# Supplementary material for: Investigation of NS3 Protease Resistance-Associated Variants and Phenotypes for the Prediction of Treatment Response to HCV Triple Therapy
Source: PLoS One. 2016 Jun 9;11(6):e0156731. doi: 10.1371/journal.pone.0156731 (PMC4900565; doi:10.1371/journal.pone.0156731)
Supplement: S1 File — (DOCX) [file pone.0156731.s001.docx]

**In vitro transcription of subgenomic HCV RNAs**

*In vitro* transcription reaction mixtures (total volume 100 µl) contained 80 mM HEPES (pH 7.5), 12 mM MgCl_2_, 2 mM spermidine, 40 mM dithiothreitol (DTT), 3.125 mM of each nucleoside triphosphate, 1 U of RNasin (Promega)/µl, 0.1 µg of plasmid DNA/µl, and 0.6 U/µl of T7 RNA polymerase (Promega). After 2 h incubation at 37°C, 0.3 U of T7 RNA polymerase/µl of reaction mixture was added and the reaction mixture was incubated over night at 37°C. Transcription was terminated by adding 1.2 U of RNase-free DNase (Promega) per µg plasmid DNA and 30 min of incubation at 37°C. RNA was extracted with acidic phenol and chloroform, precipitated with isopropanol at room temperature and dissolved in RNase-free water.

**Luciferase Measurement**

Cells were lysed in in luciferase lysis buffer (1% (v/v) Triton X-100, 10% (v/v) Gylcerol, 25 mM Glycine-Glycine (pH 7.8), 15 mM µl MgSO4, 4 mM EGTA, keep at 4 °C; freshly add 1 mM DTT just before use). For Firefly Luciferase measurement cells were washed once with PBS and lysed directly on the 96-well plate with 30µl lysis buffer per well and frozen at -80°C. Shortly before measurement lysates were allowed to thaw at RT for 30 to 60 min. *Firefly* luciferase activity was measured for 10 sec in a Mithras LB940 multimode microplate reader (Berthold Technologies, Bad Wildbad). *Firefly* assay buffer (25 mM Glycyl-Glycine (pH 7.8), 15 mM K_2_PO_4_, (pH 7.8), 15 mM MgSO_4_, 4 mM EGTA, 1 mM DTT and 2 mM ATP), supplemented with 70 μM D-luciferin was added to each well automatically prior to each measurement.
